# Electrospinning of a Copolymer PVDF-*co*-HFP Solved in DMF/Acetone: Explicit Relations among Viscosity, Polymer Concentration, DMF/Acetone Ratio and Mean Nanofiber Diameter

**DOI:** 10.3390/polym13193418

**Published:** 2021-10-05

**Authors:** Petr Filip, Jana Zelenkova, Petra Peer

**Affiliations:** Institute of Hydrodynamics, Czech Academy of Sciences, 166 12 Prague, Czech Republic; zelenkova@ih.cas.cz (J.Z.); peer@ih.cas.cz (P.P.)

**Keywords:** electrospinning, nanofibrous web, poly(vinylidene-*co*-hexafluoropropylene), *N*,*N’*-dimethylformamide, acetone, master curve

## Abstract

The process of electrospinning polymer solutions depends on many entry parameters, with each having a significant impact on the overall process and where complexity prevents the expression of their interplay. However, under the assumption that most parameters are fixed, it is possible to evaluate the mutual relations between pairs or triples of the chosen parameters. In this case, the experiments were carried out with a copolymer poly(vinylidene-*co*-hexafluoropropylene) solved in mixed *N*,*N’*-dimethylformamide (DMF)/acetone solvent for eight polymer concentrations (8, 10, 12, 15, 18, 21, 24, and 27 wt.%) and five DMF/acetone ratios (1/0, 4/1, 2/1, 1/1, 1/2). Processing of the obtained data (viscosity, mean nanofiber diameter) aimed to determine algebraic expressions relating both to viscosity and a mean nanofiber diameter with polymer concentration, as well as DMF/acetone ratio. Moreover, a master curve relating these parameters with no fitting factors was proposed continuously covering a sufficiently broad range of concentration as well as DMF/acetone ratio. A comparison of algebraic evaluation with the experimental data seems to be very good (the mean deviation for viscosity was about 2%, while, for a mean nanofiber diameter was slightly less than 10%).

## 1. Introduction

Fluoropolymers, in which fluorine atoms are directly attached to their carbon-only backbone, exhibit a series of properties, suiting their frequent application in various sectors such as transport (including aviation and electric vehicles), pharmaceutical, medical, and semiconductor industries, ion-exchange membranes for energy storage among others [1,2,3]. Their unique physicochemical properties, which include durability, stability and mechanical strength in harsh conditions, chemical inertness, biocompatibility, nontoxicity, resistance to temperature and fire, make them irreplaceable by most other polymers that cannot guarantee a similar range of useful attributes.

Polyvinylidene fluoride (PVDF) has recently drawn significant attention for its applicability in membranes (membrane desalination) [4,5], electrode binders or separator coatings in batteries [6,7], photocatalyst [8,9] and energy harvesting (conversion of mechanical vibrations into electrical energy via the direct piezoelectric effect and thermal fluctuations into electrical energy via the pyroelectric effect [10]) due to its high hydrophobicity and relatively easy processability [11]. PVDF also exhibits a high dielectric constant, and can be ferroelectric in some of its crystal phases; thus, this polymer is characterized by a spontaneous polarization in the unstrained state and is capable of re-orientating its polarization direction via an applied electric field.

In spite of the simple chemical structure of PVDF, it can exhibit five different polymorphs depending on its processing conditions [12]. The most common and stable polymorph of PVDF of the five phases is the *α*-phase. However, the *β*-phase represents the most important one due to its piezoelectric and pyroelectric properties and applications which come from the largest spontaneous polarization per unit cell of *β*-PVDF [13,14]. *β*-PVDF possesses these properties due to its well-oriented polarized structure of the all-trans planar zigzag conformation (TTTT); an illustrative picture is depicted as Figure 1 in Martins et al. [15]. The problem is that the crystalline structure of PVDF obtained by the melting and solvent evaporation method is generally the *α*-phase [16].

This obstacle can be overcome by applying electrospinning as a process, naturally forming *β*-PVDF conformation. During the process of electrospinning, a high electric field (tens of kV) causes the emanation of viscoelastic jets from the polymer solutions to a collector, where the polymeric nanofibers which are free of evaporated solvent form nanofibrous mats (for details see Refs. [17,18,19]). More elaborate electrospinning devices are being continuously developed, as seen in Ref. [20]. The application of such a high electric field changes the molecular conformation of PVDF, and the polar *β*-phase is more likely to form than *α*-phase [21]. Among the factors supporting formation of *β*-phase ranges:

- high-voltage applied to the electrospun solution [21];
- high stretching ratio of the viscoelastic jets [21] analogous to uniaxial mechanical stretching causing the α- to β-phase transition [22,23,24,25];
- rotation of the collecting drum [26];
- low crystallization temperature that arises from the low environmental temperature during electrospinning [13,15];
- rapid evaporation of the solvent [13,15].

All these factors contribute to the induction of the polar *β*-phase of PVDF nanofibers during electrospinning process, resulting in no need for post-processing treatment [13].

A proper choice of solvents is crucial for the proper electrospinning of PVDF. Bottino et al. [27] studied PVDF polymer-solvent interactions based on Hansen solubility parameters [28]. Out of 46 liquids, they determined eight of them as good, including two polar aprotic solvents: well-coordinating *N*,*N´*-dimethylformamide (DMF) and weakly coordinating acetone with boiling points of 153 °C and 56 °C, respectively. Among three ‘classical’ solvents of PVDF (all classified as good), i.e., *N*,*N´*-dimethylformamide, *N*-methylpyrrolidone, and dimethyl sulfoxide, an application of the first one results in the highest piezoelectric properties and the smallest fibre diameter [29], thus substantiating its frequent usage.

Zheng et al. [13] used mixed DMF/acetone as the solvent and showed dominant participation of the *β*-phase in electrospun nanofibrous mats. This is explained by the fact that the addition of solvent with a low boiling point accelerated the evaporation rate -of the solvent- during the electrospinning process. This higher evaporation rate might lead to a lower solidifying temperature for the electrospinning jets, which may promote nucleation and thereby the crystallization of the *β*-phase of PVDF [13].

The mixed DMF/acetone solvent favouring the presence of the *β*-phase simultaneously participates in the morphology of the resulting nanofibers. The DMF/acetone ratio strongly influences both possible (dis)appearance of beads along the nanofibers (abrupt local change of diameter) and a mean nanofibers’ diameter. The addition of acetone to DMF contributes to uniform and bead-free nanofibers owing to its volatile nature [30,31]. A positive effect of the adding of acetone to DMF was also -apart from PVDF- observed for mixtures of PVDF with polylactide [32], trifluoroethylene [33,34] and poly(methyl methacrylate) [35]. Recent work [36] has studied the influence of 10 various volatile solvents (including acetone) in combination with DMF on the morphology of PVDF nanofibrous mats.

In parallel with the presence of acetone, a purity of DMF also strongly participates in the possible appearance of singularities (beads) along the nanofibers. Uyar and Besenbacher [37] used DMF with different grades as supplied by the producers, and showed that DMF conductivity ranged within the interval 0.4–10.1 µS/cm. Using polystyrene as an electrospun polymer, it was demonstrated that there is a direct proportionality between DMF purity and the vanishing of beaded nanofibers.

The increasing participation of acetone in the mixed DMF/acetone solvent results in an increasing mean nanofiber diameter [25,38,39] caused by the higher boiling point and polarity of DMF than those of acetone [40]. However, increasing the content of acetone has its limit, as at higher values, the process of electrospinning is stopped (owing to blockage of polymer solution transport) [31].

Apparent improvements in nanofibrous mat behaviour obtained from PVDF/DMF/acetone can be obtained by using a copolymer PVDF-*co*-HFP (poly(vinylidene-*co*-hexafluoropropylene) instead of a pure PVDF, and detailed information on copolymers based on PVDF is presented in Voet et al. [41]. Practically all the properties of PVDF-only nanofibrous mats are preserved in this copolymer, including the principal attribute: dominance of the *β*-phase [42]. Moreover, plasticity of the hexafluoropropylene (HFP) group enhances the chain stability [18,43]. The increased hydrophobicity (improving e.g., membrane efficiency) results from an increase in the fluorine content [44]. In addition, the copolymer exhibits the highest dielectric constant and electroactive response, including piezoelectric, pyroelectric, and ferroelectric effects, more details e.g., in [45,46].

The electrospun PVDF-*co*-HFP nanofibers proved efficient for the preparation of battery separator membranes used in Li-ion batteries [47,48] and magnesium ion batteries [49]. Compared to PVDF nanofibrous membranes, the PVDF-*co*-HFP membranes contributed to a better stability in membrane distillation applied to seawater desalination [50,51]. This application also used the copolymer as one part of dual-layer nanofibrous membranes with promising results [52], while PVDF-co-HFP nanofibrous mats also showed their applicability in passive ice protection [53]. The behaviour of electrospun copolymer PVDF-*co*-HFP (20 wt.% and 15 wt.%) solved in DMF/acetone in the ratio 4/1 was studied in Shahabadi [54] and Zhao et al. [55], respectively.

As analysis of this behaviour of electrospun polymer solution is rather scarce in comparison with the application of only pure DMF, the aim of this contribution is to determine the functional relations expressing a mean nanofiber diameter through used concentration of the copolymer PVDF-*co*-HFP and a ratio DMF/acetone, and viscosity in dependence on the same variables. For every application of nanofibrous mats, the mean nanofiber diameter plays a decisive role in that specific use-case and where each application has its specific diameter range. For this reason, being able to predict the resultant diameter range dependent on the entry parameters attracts the corresponding attention. Indeed, some entry parameters can pre-determine the possible suitability of the polymer solutions when electrospun. The viscosity parameter represents one of the key influencing factors predetermining a passage from electrospinning to electrospraying, the creation of blobs and even electrospinning suppression, but especially significantly participates in obtaining the range of nanofibrous diameters. Hence, the interplay between these two characteristics (viscosity and diameter) is of crucial importance. As a main result, a master curve determining viscosity of the polymer solution is presented. In other words, polymer solution viscosity can be determined by using only a ratio of DMF/acetone and polymer concentration. In this relation, no fixing parameters are involved and this estimate is continuously valid over a sufficiently broad region of concentration and DMF/acetone ratio. Validity of the proposed relations are confirmed by the experiments carried out in the concentration region 8–27 wt.% (8 discrete values: 8, 10, 12, 15, 18, 21, 24, and 27 wt.%) and DMF/acetone ratio 1/0–1/2 (5 discrete values: 1/0, 4/1, 2/1, 1/1, and 1/2). Correspondence between theoretical predictions and the experimental data is very good.

## 2. Materials and Methods

### 2.1. Material

**Figure 1:**
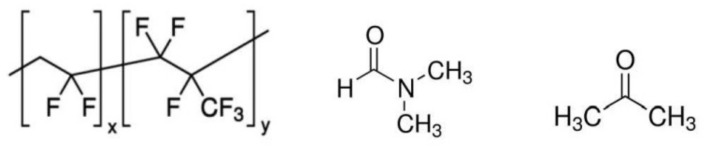
Chemical structures (from left to right) of PVDF-*co*-HFP, DMF and acetone.

Kynar Flex^®®^ 2801 (copolymer poly(vinylidene fluoride)-*co*-hexafluoropropylene), *M*_w_ = 455,000 g/mol, datasheet [56], was purchased from Arkema (Colombes, France), *N*,*N´*-dimethylformamide (DMF) (p.a., >99.5%) was purchased from P-LAB, a.s. (Prague, Czech Republic), acetone was purchased from Sigma-Aldrich (Merck, St. Louis, MO, USA), purity > 99% (by HPLC quality). All chemicals were used as obtained without further refinement. The chemical structures are depicted in Figure 1.

### 2.2. Preparation of Electrospinning Solution

The copolymer PVDF-*co*-HFP was dissolved in five ratios of the mixed DMF/acetone solvents (pure DMF 1/0, 4/1, 2/1, 1/1, 1/2) using a magnetic stirrer MR Hei-Tec (Heidolph Instruments GmbH, Schwabach, Germany) with the help of a teflon-coated magnetic cross under these conditions: mixing rate was 250 rpm, temperature 25 °C and time of mixing was 24 h. The content of copolymer was successively changed (eight concentrations): 8, 10, 12, 15, 18, 21, 24, and 27 wt.%. The higher concentrations in combination with DMF/acetone ratio of 1/2 were not completely applicable due to poor mixing and the inability to electrospin these materials as discussed later (high viscosity, suppression of Taylor’s cones). 

### 2.3. Process of Electrospinning

For the process of electrospinning we used our laboratory needleless device equipped with a high-voltage power supply SL70PN150 (Spellman, Hauppauge, NY, USA), a carbon steel stick (10 mm in diameter) with a semispherical hole for depositing 0.2 mL of polymer solution and a motionless, flat metal collector, for details see Ref. [57]. The basic parameters were set to the values: a voltage of 18 kV, the fixed tip-to-collector distance of 100 mm, temperature of 21 ± 3 °C, a relative humidity of 35 ± 5%.

### 2.4. Rheological Measurements

A rotational rheometer Physica MCR 501 (Anton Paar, Graz, Austria) equipped with the concentric cylinder geometry (the inner and outer diameters were 26.6 and 28.9 mm, respectively) was used both for oscillatory measurements (frequency sweep within 0.1–100 Hz at strain 1%) providing elatic *G*’ and viscous *G*” moduli, and for shear viscosity measurements (a range 0.01–300 s^−1^). The value of shear rate $\dot{\gamma }$ = 0.12 s^−1^ belonging to a linear viscoelastic region was chosen for measurement of shear viscosity of copolymer solutions with different polymer concentrations and DMF/acetone ratios. Temperature was set to 25 °C. Each measurement was carried out at least three times and the individual runs were practically identical.

### 2.5. Characterization of Nanofibrous Mats

A high-resolution scanning electron microscope Vega 3 (Tescan, Brno, Czech Republic) was used for nanofibrous mats characterization. First, the samples were sputtered by a conductive coating layer using a sputter Quorum Q150R (Quorum Technologies Ltd., Laughton, UK). A mean nanofiber diameter derived from 300 measurements taken from three different images was determined by applying the Adobe Creative Suite software (San Jose, CA, USA).

## 3. Results and Discussion

As already stated above, both DMF and acetone are good solvents of PVDF-*co*-HFP. This is confirmed by the Hansen solubility parameters [28] summarized in Table 1 including -for a comparison- also data for pure PVDF. The data for PVDF and PVDF-*co*-HFP was taken from Bottino et al. [27] and Meringolo et al. [58], respectively. The individual Hansen solubility parameters (HSP) have the following meaning:

*δ*_d_-the energy density from dispersion bonds between molecules; 

*δ*_p_-the energy from dipolar intermolecular force between molecules;

*δ*_h_-the energy from hydrogen bonds between molecules.

If the HSP parameters are relatively close, i.e., a distance *R*_a_ (where Δ*δ* is a difference between the corresponding parameters)

(1) $$R_{a}=\sqrt{4{\left(\Delta \delta_{d}\right)}^{2}+{\left(\Delta \delta_{p}\right)}^{2}+{\left(\Delta \delta_{h}\right)}^{2}}$$

is relatively low, then two components are mutually solvable.

**Figure 2:**
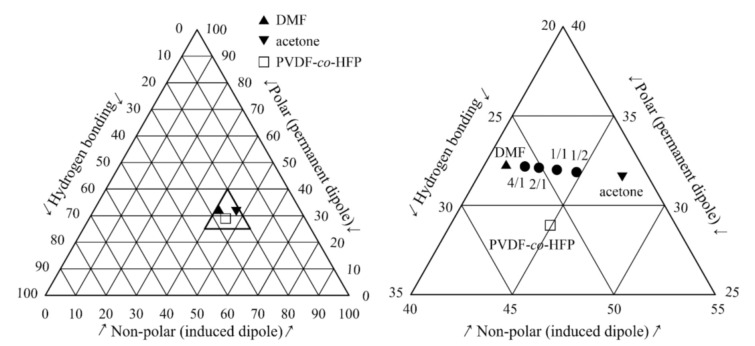
The Teas diagram relating locations of PVDF-*co*-HFP, DMF and acetone (**left**) and its magnification (**right**) also introduce the individual solvent mixtures.

The so-called Teas diagram is presented in Figure 2. This diagram introduces percentual participation of all HSP parameters and provides a relatively good insight into the mutual location of the copolymer and the individual solvents with DMF/acetone ratio 1/0 (pure DMF), 4/1, 2/1, 1/1, 1/2, and 0/1 (pure acetone).

**Figure 3:**
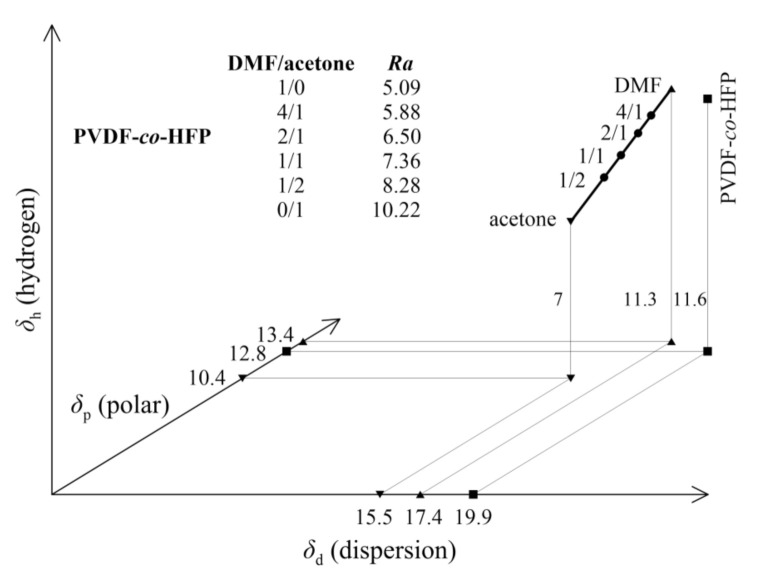
The Hansen space with location of the individual solvents and PVDF-*co*-HFP.

The Teas diagram projects three HSP parameters onto a 2D chart, however the distances between the individual solvents and PVDF-*co*-HFP are more apparent with a graphical presentation in the so-called Hansen space, see Figure 3. Here, we can observe an increasing distance of the solvent mixture with an increasing content of acetone from PVDF-*co*-HFP. 

**Figure 4:**
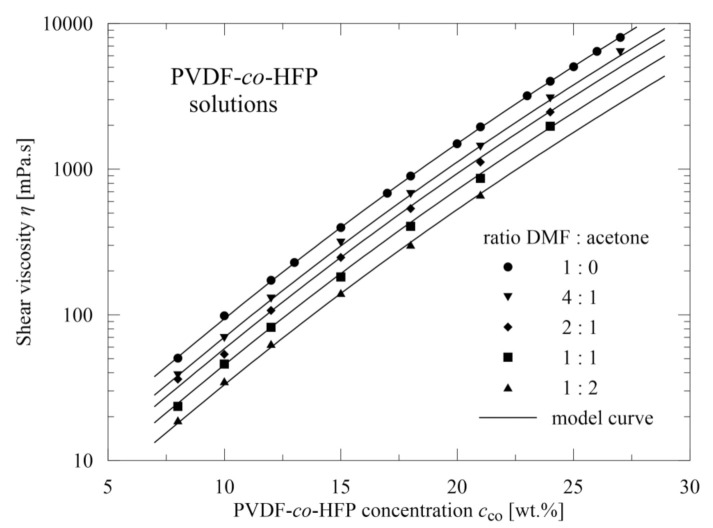
Viscosity measurements for various copolymer concentrations and DMF/acetone ratios.

In evaluating the polymeric materials, viscosity was measured as depicted in Figure 4. Based on oscillatory measurements, the value of $\dot{\gamma }$ = 0.12 s^−1^ was chosen within the linear viscoelastic region. As expected, for fixed copolymer concentration *c*_co_ [wt.%], the viscosity decreases with increasing participation of acetone *c*_ac_ [wt.%] in the solvent mixture. However, for higher values of *c*_co_ we can see that no data are at disposal, due to the fact that in this case mixture behaviour rapidly deteriorates and viscosity cannot be determined, as well as no nanofibers being able to be electrospun (which is also confirmed in the literature [30]).

Relatively dense covering of concentrations and DMF/acetone ratios gives a possibility to express the mutual dependence of viscosity *η* on copolymer concentration *c*_co_ and acetone concentration *c*_ac_ in the solvent mixture (0, 20, 33.3, 50, 66.6, and 100 wt.%)

(2) $$\eta =\left(9.4-0.23\;c_{ac}^{0.78}\right)\times 10^{log^{3}c_{co}}\;$$

or in a simpler form

(3) $$\log(\eta )=log^{3}c_{co}+log\left(9.4-0.23\;c_{ac}^{0,78}\right)$$

Here we can see that the deviations of the experimental points from the prediction are practically negligible, as seen in Figure 4. Further, the expression (3) is composed of two separate terms: the first one, depending on the copolymer concentration only, and the second one depending only on acetone participation in the mixture solvent. This indicates how viscosity can be altered and thus, as viscosity significantly influences the quality of the resulting nanofibrous mats, how to achieve the required nanofibers. It should be emphasized that the relation (3) is continuously valid for all combinations of *c*_co_ and *c*_ac_, including the combinations not used in the experiments, and no additional fitting parameters are required. 

The relation (3) can be transformed to a master curve using the transformed viscosity *η*_trans_

(4) $$\eta_{trans}=\eta /\left(9.4-0.23\;c_{ac}^{0,78}\right)\;$$

**Figure 5:**
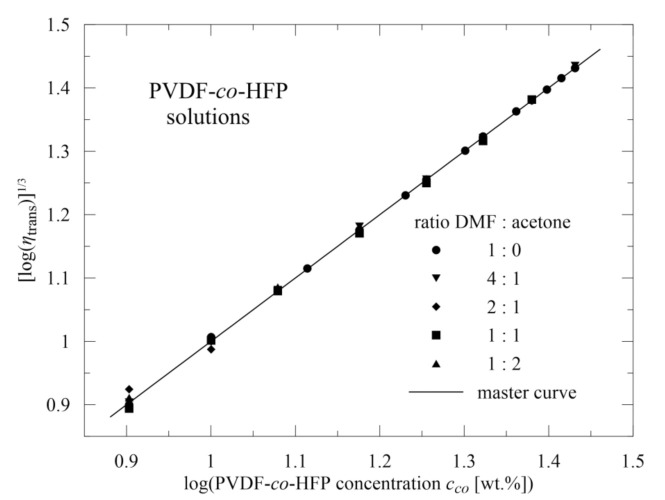
Master curve of transformed viscosity.

The master curve is of the form

(5) $${\left[\log\left(\eta_{trans}\right)\right]}^{1/3}=\log c_{\mathrm{co}}$$

and as apparent from Figure 5, represents an axis of the first quadrant in the corresponding coordinates. It should be again emphasized that no additional fitting parameter is required and coincidence across all experimental data is very good.

**Figure 6:**
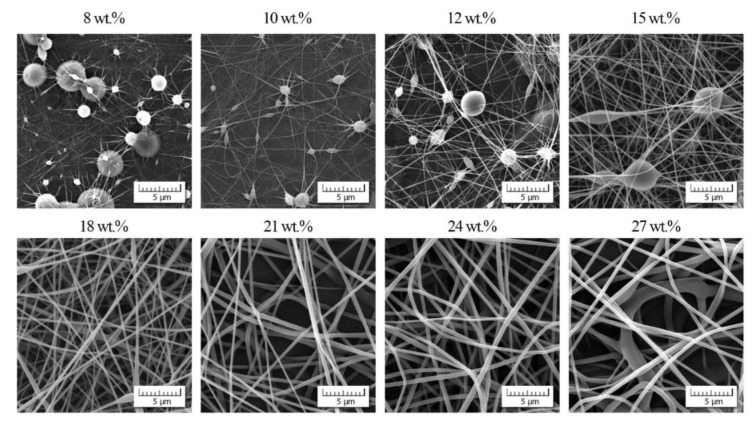
Scanning electron micrographs (SEM) showing the morphology of individual nanofibers for various copolymer concentrations and DMF/acetone ratio 4/1.

**Figure 7:**
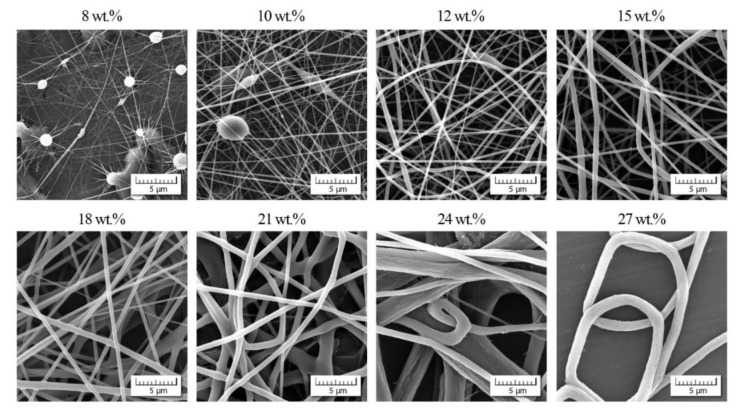
Scanning electron micrographs (SEM) showing the morphology of individual nanofibers for various copolymer concentrations and DMF/acetone ratio 1/1.

Simultaneously with viscosity measurements, the process of electrospinning was applied with the same range of the copolymer concentration and DMF/acetone ratios; for illustration, see Figure 6 and Figure 7.

**Figure 8:**
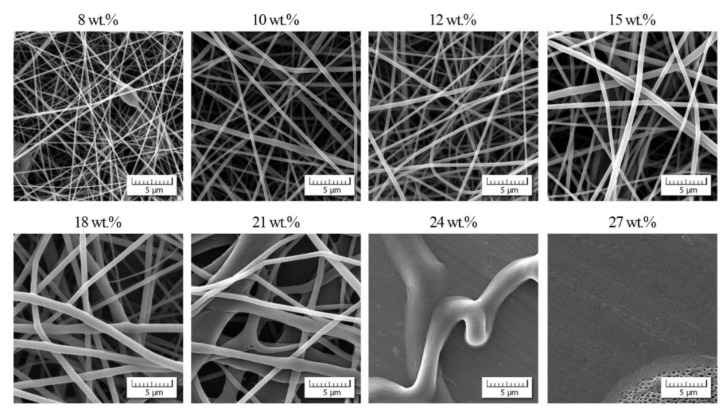
Scanning electron micrographs (SEM) showing the morphology of individual nanofibers for various copolymer concentrations and DMF/acetone ratio 1/2.

In these two examples, we can observe two phenomena: (1) suppression of bead appearance with increasing participation of acetone as already introduced in the Introduction, (2) gradual passage to non-acceptable nanofibrous quality for higher values of *c*_ac_ as confirmed in Figure 8.

This indicates that the positive impact of acetone is lost for higher *c*_ac_ and higher copolymer concentrations of *c*_co_.

**Figure 9:**
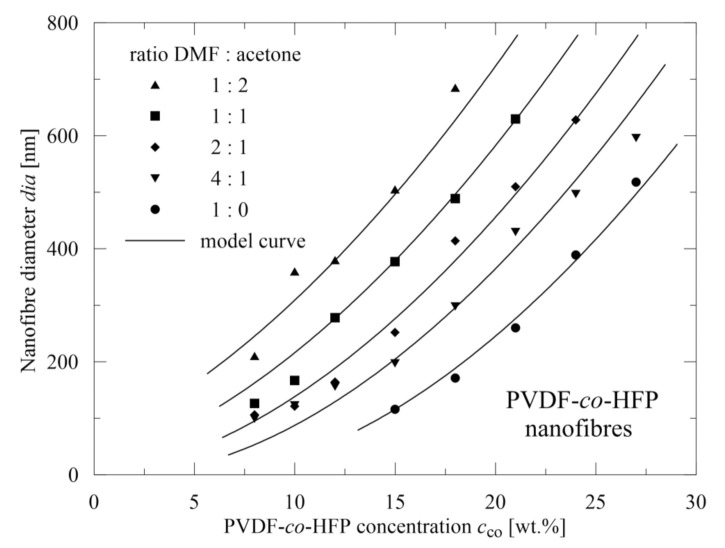
A course of a mean nanofibers diameter and its evaluation.

Based on 40 combinations of *c*_co_ (8) and *c*_ac_ (5), it was possible to approximate a course of a mean nanofiber diameter *dia* [nm] in dependence on these two variables, see Figure 9. The omitted points represent the situations when no nanofibrous mats could be electrospun (higher *c*_co_ and *c*_ac_). The proposed relation is of the form

(6) $$dia=1.82\times {\left(-5.2+c_{\mathrm{co}}+0.18\;c_{\mathrm{ac}}\right)}^{1.82}\;$$

As accuracy in determining a nanofiber diameter is not comparable with that of viscosity, the mean deviation of experimentally determined (measured using SEM pictures by means of Adobe Creative Suite software (San Jose, CA, USA)) mean diameters differs from the predicted ones by 9.9%. However, as in the preceding case, this relation (6) can be applied continuously across the whole range of copolymer concentrations and acetone participation. Nevertheless, validity of this relation is limited by higher values of *c*_co_ and *c*_ac_ as discussed earlier. 

Continuous validity of both relations for determining viscosity and nanofiber diameter gives the possibility of optimizing the process of electrospinning in the sense that it is possible to prepare tailor-made nanofibrous quality.

Remark: Experimental data sets are summarized in Supplementary Material.

## 4. Conclusions

A sufficiently broad range of selected PVDF-*co*-HFP concentrations and DMF/acetone ratios makes it possible to derive the functional dependencies of the copolymer viscosity and electrospun nanofiber diameters on the copolymer concentration and the DMF/acetone ratio. With an increasing percentage of acetone, the quality of nanofibrous mats further improves (reducing unwanted beads along the nanofibers). However, this positive phenomenon is limited for higher polymer concentrations (at about 25 wt.% and more) when high concentrations and high contents of acetone are combined (where the DMF/acetone ratio is lower than approximately 1/2) this will prevent a copolymer solution from being electrospun (due to the higher viscosity). If nanofibers of a specific mean diameter are required, the obtained algebraic relations make it possible to choose a corresponding concentration and DMF/acetone ratio. Since a continuous series of such pairs is available, it is possible to decide which one is optimal for an application (e.g., no bead formation). Such an approach will eliminate the traditional trial-and-error method, saving time and cost.

## Figures and Tables

**Table 1:** Summary of HSP parameters for entry components.

Component	*δ*_d_ [MPa^1/2^]	*δ*_p_ [MPa^1/2^]	*δ*_h_ [MPa^1/2^]
PVDF	17.2	12.5	9.2
PVDF-*co*-HFP	19.9	12.8	11.6
DMF	17.4	13.7	11.3
acetone	15.5	10.4	7.0

## Supplementary Materials

The following are available online at https://www.mdpi.com/article/10.3390/polym13193418/s1, MS Excel file: Experimental Data Sets.xlsx.
