# Supplementary material for: Electrospinning of a Copolymer PVDF-co-HFP Solved in DMF/Acetone: Explicit Relations among Viscosity, Polymer Concentration, DMF/Acetone Ratio and Mean Nanofiber Diameter
Source: Polymers (Basel). 2021 Oct 5;13(19):3418. doi: 10.3390/polym13193418 (PMC8512270; doi:10.3390/polym13193418)
Supplement: Supplementary file 1 [file polymers-13-03418-s001.zip › polymers-1402519-supplementary.pdf]

experiments

Concentration wt.%	DMF:AC 1:0 eta exp mPa.s	DMF:AC 4:1 eta exp mPa.s	DMF:AC 2:1 eta exp mPa.s	DMF:AC 1:1 eta exp mPa.s	DMF:AC 1:2 eta exp mPa.s
8	50,35	38,5	36,1	23,5	18,9
10	98,47	69,4	53,6	45,8	35
12	172,21	129	107	82	63
13	228,52				
15	397,41	314	248	182	141
17	684,48				
18	897,12	673,2	537	406	303
20	1497,85				
21	1950,23	1429	1117	865	669
23	3195,85				
24	4013,25	3059	2470	1966	
25	5034,43				
26	6425,25				
27	8021,75	6355			

experiments

Concentration wt.%	DMF:AC 1:0 dia nm	DMF:AC 4:1 dia nm	DMF:AC 2:1 dia nm	DMF:AC 1:1 dia nm	DMF:AC 1:2 dia nm
8		99	106	126	210
10		123	121	167	360
12		156	164	278	380
15	116	197	252	377	505
18	171	298	414	489	685
21	260	430	510	630	1123
24	389	497	628	1295	3485
27	518	596		1440	
